# Supplementary material for: The Complete Chloroplast Genome Sequencing and Comparative Analysis of Reed Canary Grass (Phalaris arundinacea) and Hardinggrass (P. aquatica)
Source: Plants (Basel). 2020 Jun 14;9(6):748. doi: 10.3390/plants9060748 (PMC7356517; doi:10.3390/plants9060748)
Supplement: Supplementary file 1 [file plants-09-00748-s001.zip › Table S1.docx]

Table S1. Sampled Gramineae species along with their Genebank accession codes of cp genomes in this study.

| Species | GenBank accession code |
| --- | --- |
| *Phleum alpinum* | NC027482.1 |
| *Alopecurus arundinaceus* | NC037163.1 |
| *Hierochloe odorata* | NC027475.1 |
| *Anthoxanthum odoratum* | NC027467.1 |
| *Triticum aestivum* | KC912694.1 |
| *Triticum monococcum* | NC021760.1 |
| *Triticum Urartu* | NC021762.1 |
| *Avena sativa* | NC027468.1 |
| *Bambusa multiplex* | NC024668.1 |
| *Panicum virgatum* | HQ731441.1 |
| *Lolium perenne* | NC009950.1 |
| *Lolium multiflorum* | JX871942.1 |
