# Supplementary material for: The Complete Chloroplast Genome Sequencing and Comparative Analysis of Reed Canary Grass (Phalaris arundinacea) and Hardinggrass (P. aquatica)
Source: Plants (Basel). 2020 Jun 14;9(6):748. doi: 10.3390/plants9060748 (PMC7356517; doi:10.3390/plants9060748)
Supplement: Supplementary file 1 [file plants-09-00748-s001.zip › Table S2.docx]

Table S2. Summary of polymorphisms detected among three *Phalaris* cp genomes.

|  | *P. aquatica* (4x) vs. *P. arundinacea* (6x) | | | | | *P. aquatica* (4x) vs. *P. arundinacea* (4x) | | | | | *P. arundinacea* (4x) vs. *P. arundinacea* (6x) | | | | |
| --- | --- | --- | --- | --- | --- | --- | --- | --- | --- | --- | --- | --- | --- | --- | --- |
| Gene | In/Del | Tn | Tv | Nonsyn | Total | In/Del | Tn | Tv | Nonsyn | Total | In/Del | Tn | Tv | Nonsyn | Total |
| *atpB* |  |  | 3 |  | 3 |  |  | 3 | 1 | 3 |  |  |  |  |  |
| *atpI* |  |  | 3 |  | 3 |  |  | 3 |  | 3 |  |  |  |  |  |
| *ccsA* |  |  | 1 | 1 | 1 |  |  | 1 | 1 | 1 |  |  |  |  |  |
| *cemA* |  |  | 2 |  | 2 |  |  | 2 |  | 2 |  |  |  |  |  |
| *clpP* |  |  | 1 |  | 1 |  |  | 1 |  | 1 |  |  |  |  |  |
| *infA* |  |  | 3 | 1 | 3 |  |  | 3 | 1 | 3 |  |  |  |  |  |
| *matK* |  | 3 | 8 | 7 | 11 |  | 3 | 8 | 7 | 11 |  |  |  |  |  |
| *ndhA* |  | 1 | 3 | 2 | 4 |  | 1 | 3 | 2 | 4 |  |  |  |  |  |
| *ndhD* |  |  | 1 | 1 | 1 | — | — | — | — | — |  |  |  |  |  |
| *ndhF* |  |  | 3 | 2 | 3 |  |  | 2 | 1 | 2 |  |  |  |  |  |
| *ndhH* |  |  | 7 |  | 7 |  |  | 7 |  | 7 |  |  |  |  |  |
| *ndhI* |  | 1 | 1 | 1 | 2 |  | 1 | 1 | 1 | 2 |  |  |  |  |  |
| *ndhK* |  | 1 | 4 | 1 | 5 |  | 1 | 4 | 1 | 5 |  |  |  |  |  |
| *petA* |  |  | 3 |  | 3 |  |  | 3 |  | 3 |  |  |  |  |  |
| *petB* |  |  | 2 |  | 2 |  |  | 2 |  | 2 |  |  |  |  |  |
| *psaA* |  |  | 2 |  | 2 |  |  | 3 |  | 3 |  |  |  |  |  |
| *psaB* |  |  | 3 |  | 3 |  |  | 3 |  | 3 |  |  |  |  |  |
| *psaC* | 2 |  | 1 |  | 3 | 2 |  | 1 |  | 3 |  |  |  |  |  |
| *psbA* |  |  | 1 |  | 1 |  |  | 1 |  | 1 |  |  |  |  |  |
| *psbB* |  |  | 1 |  | 1 |  |  | 1 |  | 1 |  |  |  |  |  |
| *psbC* |  |  | 4 |  | 4 |  |  | 4 |  | 4 |  |  |  |  |  |
| *psbD* |  |  | 3 |  | 3 |  |  | 3 |  | 3 |  |  |  |  |  |
| *psbH* |  |  | 1 | 1 | 1 |  |  | 1 | 1 | 1 |  |  |  |  |  |
| *psbK* | 1 |  |  |  | 1 | 1 |  |  |  | 1 |  |  |  |  |  |
| *psbZ* |  |  | 1 |  | 1 |  |  | 1 |  | 1 |  |  |  |  |  |
| *rbcL* | 1 |  | 4 |  | 5 | 1 |  | 4 |  | 5 |  |  |  |  |  |
| *rpl14* |  | 1 |  |  | 1 |  | 1 |  |  | 1 |  |  |  |  |  |
| *rpl16* |  | 2 | 3 | 3 | 5 |  | 2 | 3 | 3 | 5 |  |  |  |  |  |
| *rpl20* |  | 1 |  |  | 1 |  | 1 |  |  | 1 |  |  |  |  |  |
| *rpl22* |  |  | 1 |  | 1 |  |  | 1 |  | 1 |  |  |  |  |  |
| *rpl23* |  |  | 2 |  | 2 |  |  | 2 |  | 2 |  |  |  |  |  |
| *rpl32* | 1 |  |  |  | 1 | 1 |  |  |  | 1 |  |  |  |  |  |
| *rpl33* |  | 1 |  | 1 | 1 |  | 1 |  | 1 | 1 |  |  |  |  |  |
| *rpl36* |  |  | 1 |  | 1 |  |  | 1 |  | 1 |  |  |  |  |  |
| *rpoA* |  |  | 7 | 2 | 7 |  |  | 7 | 2 | 7 |  |  |  |  |  |
| *rpoB* | 1 | 3 | 9 |  | 13 | 1 | 3 | 9 |  | 13 |  |  |  |  |  |
| *rpoC1* |  |  | 1 |  | 1 |  |  | 1 |  | 1 |  |  |  |  |  |
| *rpoC2* |  | 2 | 27 | 13 | 29 |  | 2 | 26 | 13 | 28 |  |  |  |  |  |
| *rps11* |  |  | 1 |  | 1 |  |  | 1 |  | 1 |  |  |  |  |  |
| *rps18* |  |  | 1 |  | 1 |  |  | 2 |  | 2 |  |  |  |  |  |
| *rps2* |  |  | 1 |  | 1 |  |  | 1 |  | 1 |  |  |  |  |  |
| *rps3* |  |  | 2 | 1 | 2 |  |  | 2 | 1 | 2 |  |  |  |  |  |
| *rps4* |  | 1 |  |  | 1 |  | 1 |  |  | 1 |  |  |  |  |  |
| *rps8* |  |  | 2 | 1 | 2 |  |  | 2 | 1 | 2 |  |  |  |  |  |
| Subtotal coding | 6 | 17 | 124 | 38 | 147 | 6 | 17 | 123 | 37 | 146 |  |  |  |  |  |
| Subtotal noncoding | 92 | 68 | 275 | — | 435 | 89 | 60 | 274 | — | 423 | 14 | 7 | 10 | — | 31 |
| Total | 98 | 85 | 399 | 38 | 582 | 95 | 77 | 397 | 37 | 569 | 14 | 7 | 10 | — | 31 |

Note: Tn, Transition; Tv, Transversion; In/Del, insertion or deletion
