# Supplementary material for: The Complete Chloroplast Genome Sequencing and Comparative Analysis of Reed Canary Grass (Phalaris arundinacea) and Hardinggrass (P. aquatica)
Source: Plants (Basel). 2020 Jun 14;9(6):748. doi: 10.3390/plants9060748 (PMC7356517; doi:10.3390/plants9060748)
Supplement: Supplementary file 1 [file plants-09-00748-s001.zip › Table S3.docx]

Table S3. Large Indels among three *Phalaris* species/ploidies.

| Insertion | Position | Length (bp) | Location | Sequence |
| --- | --- | --- | --- | --- |
| *P. aquatica* (4x)  vs.  *P. arundinacea* (4x) | 4088—4116^A^ | 29 | *matK*—*tRNA-UUG* | aggttagagcgactccaatcacgatgtaa |
|  | 6737—6738^A^ | 2 | *psbK* | ga |
|  | 20623—20626^A^ | 4 | *rpoB* | gaaa |
|  | 31600—31634^B^ | 35 | *atpI*—*atpH* | ttacttctccccaatagagatagagcttagaggta |
|  | 55595—55616^A^ | 22 | *rbcL* | taactagataaaactaaatata |
|  | 55630—55654^A^ | 25 | *rbcL*—*psaI* | taaaaaataaaagaaataaaaagag |
|  | 58256—58478^A^ | 223 | *ycf4*—*cemA* | tttttttttttcaa------aaaggtc |
|  | 62087—62111^B^ | 25 | *psaI*—*petL* | tgaggtgaggtcctacttattttca |
|  | 70479—70494^B^ | 16 | *psbH*—*petB* | aaaaccaggaagtcat |
|  | 101426—101468^A^ | 43 | *rps15*—*ndhF* | tttgcttattttattagtagtcttgcttattttattagtagtc |
|  | 104051—104067^B^ | 17 | *ndhF*—*rpl32* | ctaaataaggatattta |
|  | 105166—105193^A^ | 28 | *rpl32* | aaacaaaagggtttttcggggcaacaac |
|  | 108730—108731^B^ | 2 | *psaC* | at |
|  | 109064—109082^A^ | 19 | *psaC* | aattagtactatcatgagt |
|  | 114823—114865^A^ | 43 | *ndhH*—*rps15* | taagactactaataaaataagcaagactactaataaaataagc |
| *P. aquatica* (4x)  vs.  *P. arundinacea* (6x) | 4088—4116^A^ | 29 | *matK*—*tRNA-UUG* | aggttagagcgactccaatcacgatgtaa |
|  | 6737—6738^A^ | 2 | *psbK* | ga |
|  | 20623—20626^A^ | 4 | *rpoB* | gaaa |
|  | 31609—31643^C^ | 35 | *atpI*—*atpH* | ttacttctccccaatagagatagagcttagaggta |
|  | 55595—55616^A^ | 22 | *rbcL* | taactagataaaactaaatata |
|  | 55630—55654^A^ | 25 | *rbcL*—*psaI* | taaaaaataaaagaaataaaaagag |
|  | 58256—58478^A^ | 223 | *ycf4*—*cemA* | tttttttttttcaa------aaaggtc |
|  | 62098—62122^C^ | 25 | *psaI*—*petL* | tgaggtgaggtcctacttattttca |
|  | 70491—70506^C^ | 16 | *psbH*—*petB* | aaaaccaggaagtcat |
|  | 101426—101468^A^ | 43 | *rps15*—*ndhF* | tttgcttattttattagtagtcttgcttattttattagtagtc |
|  | 104063—104079^C^ | 17 | *ndhF*—*rpl32* | ctaaataaggatattta |
|  | 105166—105193^A^ | 28 | *rpl32* | aaacaaaagggtttttcggggcaacaac |
|  | 108742—108743^C^ | 2 | *psaC* | at |
|  | 109064—109082^A^ | 19 | *psaC* | aattagtactatcatgagt |
|  | 114823—114865^A^ | 43 | *ndhH*—*rps15* | taagactactaataaaataagcaagactactaataaaataagc |
| *P. arundinacea* (4x)  vs.  *P. arundinacea* (6x) | 1282—1283^C^ | 2 | *psbA*—*tRNA-UUU* | at |
|  | 7280—7281^C^ | 2 | *psbK*—*psbI* | ta |
|  | 7482—7483^B^ | 2 | *psbI*—*tRNA-GCU* | at |
|  | 11113—11118^C^ | 5 | *psbC*—*tRNA-UGA* | gttttt |
|  | 16371—16373^C^ | 3 | *tRNA-GUC*—*psbM* | caa |
|  | 17684—17685^B^ | 2 | *petN*—*tRNA-GCA* | ga |
|  | 31183—31185^C^ | 3 | *atpI*—*atpH* | att |
|  | 41189—41190^C^ | 2 | *psaA*—*tRNA-GGA* | ga |
|  | 47175—47176^B^ | 2 | *tRNA-UAA*—*tRNA-GAA* | ac |
|  | 55713—55714^C^ | 2 | *rbcL*—*cemA* | ct |
|  | 56285—56286^B^ | 2 | *rbcL*—*cemA* | at |
|  | 60725—60727^C^ | 3 | *petA*—*psbJ* | aaa |
|  | 62618—62620^C^ | 3 | *psaI*—*petL* | att |
|  | 64253—64254^B^ | 2 | *psaJ*—*rpl33* | ga |

Note: ^A^Position numbers refer to the cp genome of *P. aquatica* (Tetraploid, 4x); ^B^Position numbers refer to the cp genome of *P. arundinacea* (Tetraploid, 4x); ^C^Position numbers refer to the cp genome of *P. arundinacea* (Hexaploid, 6x) cp genome; The listed Indels were filtered with the criterion that more than 15bp length, or occurs at the coding regions.
