# Supplementary material for: The Complete Chloroplast Genome Sequencing and Comparative Analysis of Reed Canary Grass (Phalaris arundinacea) and Hardinggrass (P. aquatica)
Source: Plants (Basel). 2020 Jun 14;9(6):748. doi: 10.3390/plants9060748 (PMC7356517; doi:10.3390/plants9060748)
Supplement: Supplementary file 1 [file plants-09-00748-s001.zip › Table S6.docx]

| Species/ploidies | TRE | REG | REN | RENG |
| --- | --- | --- | --- | --- |
| *P. aquatica* (4x) | 407 | 214 (52.58%) | 361 (88.70%) | 173 (80.84%) |
| *P. arundinacea* (6x) | 83 | 56 (67.47%) | 23 (27.71%) | 15 (26.79%) |
| *P. arundinacea* (4x) | 76 | 54 (71.05%) | 19 (25.00%) | 12 (22.22%) |

Table S6. Transcript diversity revealed by RNA editing (RE).

Note: TRE, Total RE sites. REG, RE in genic region. REN, RE caused nonsynonymous. RENG, RE caused nonsynonymous in genic region
