# Supplementary figures and images for: The Complete Chloroplast Genome Sequencing and Comparative Analysis of Reed Canary Grass (Phalaris arundinacea) and Hardinggrass (P. aquatica)
Source: Plants (Basel). 2020 Jun 14;9(6):748. doi: 10.3390/plants9060748 (PMC7356517; doi:10.3390/plants9060748)

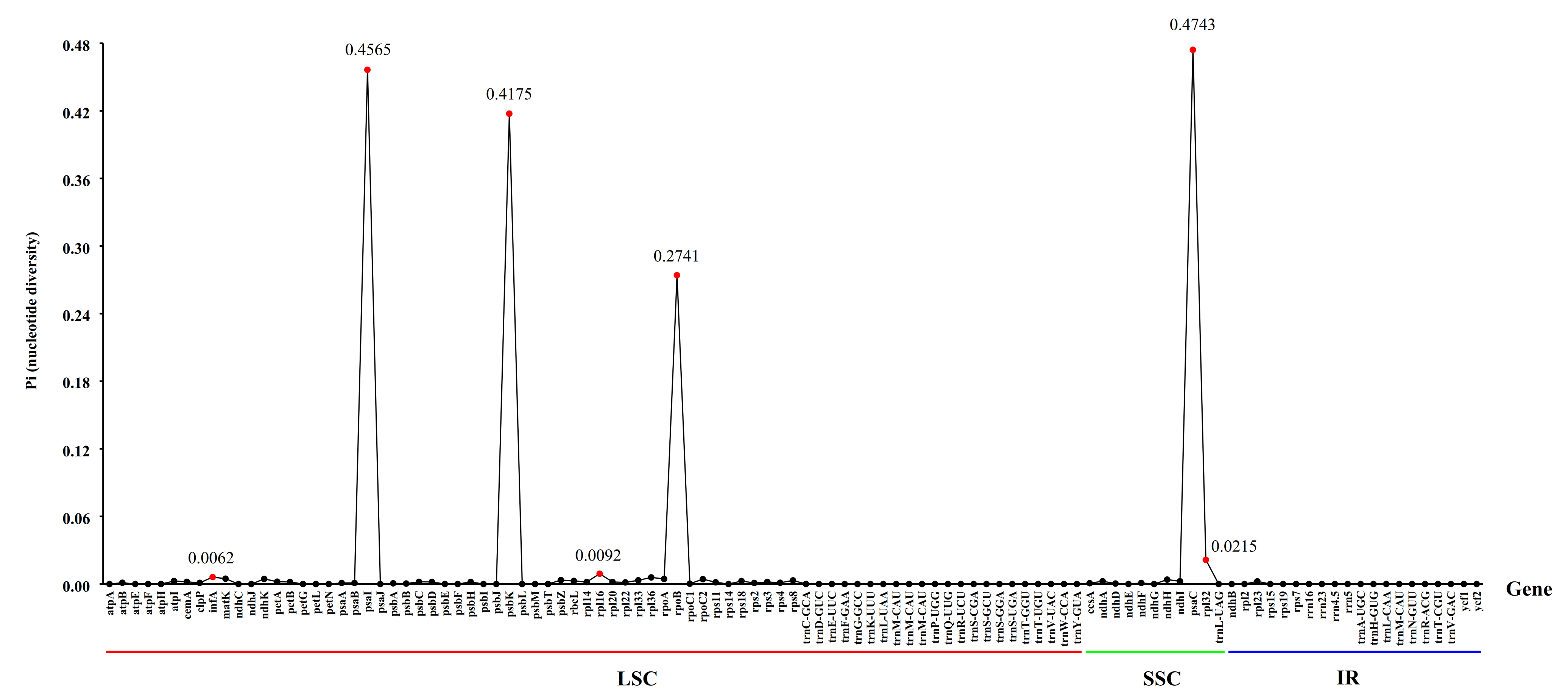

Supplement: Supplementary file 1 [file plants-09-00748-s001.zip › Fig S2.tif]

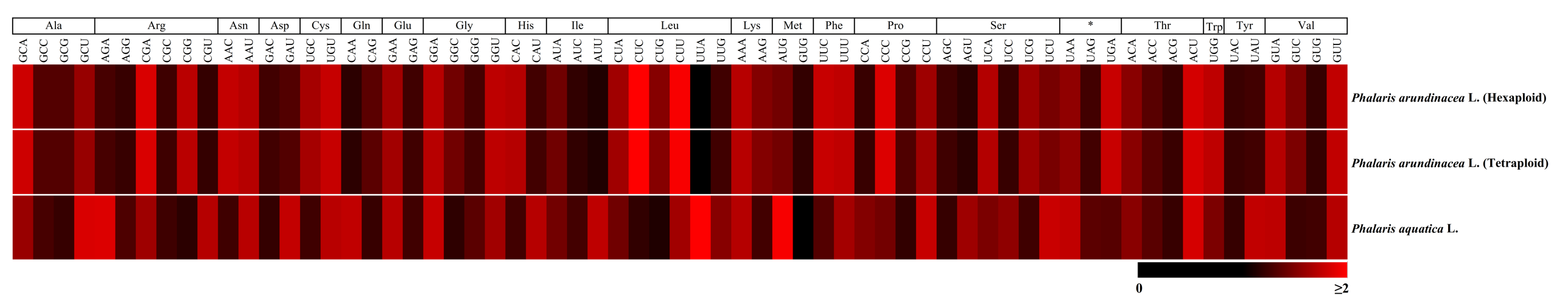

Supplement: Supplementary file 1 [file plants-09-00748-s001.zip › Fig S3.tif]

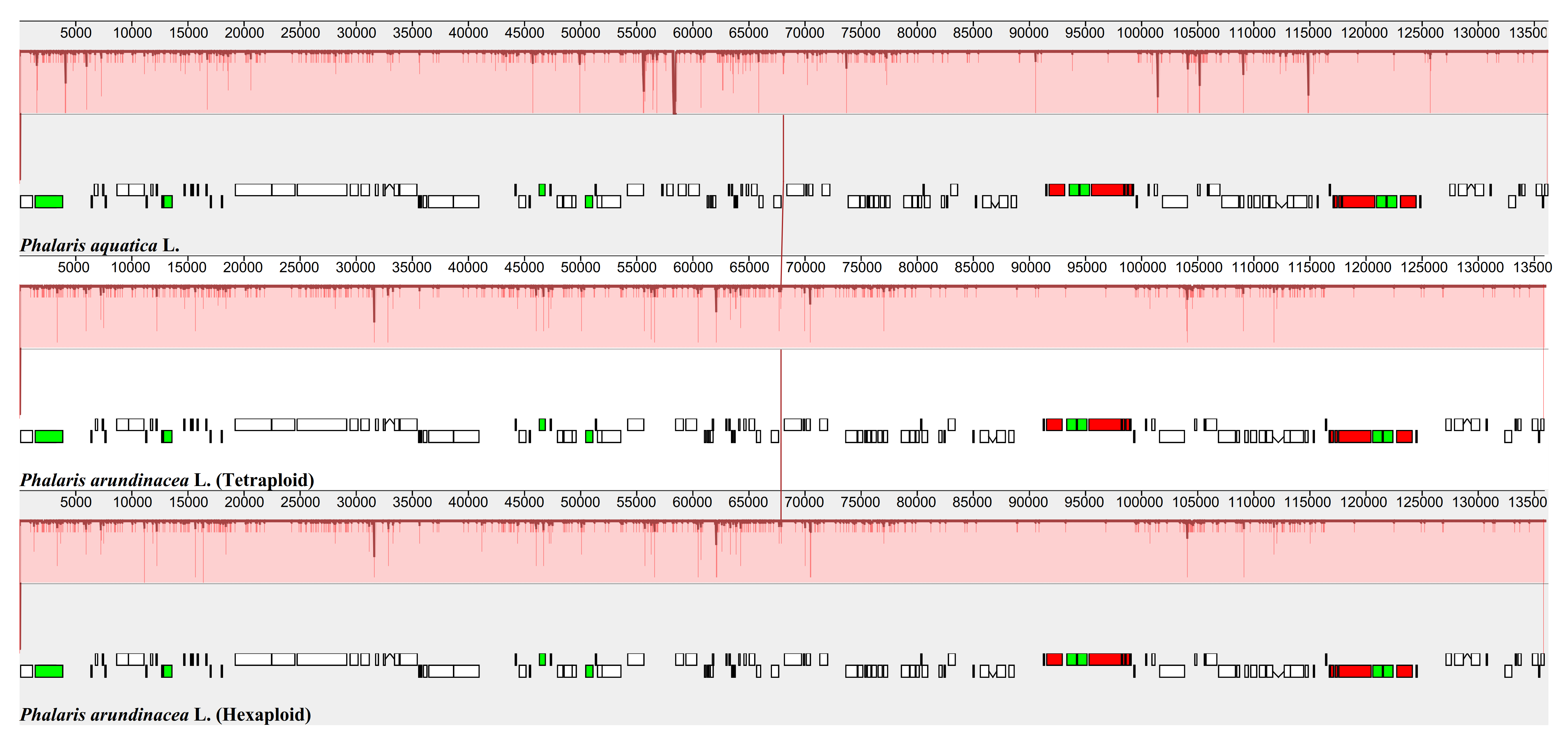

Supplement: Supplementary file 1 [file plants-09-00748-s001.zip › Fig S1.tif]
